# A population-based study of mortality due to muscular dystrophies across a 36-year period in Spain

**DOI:** 10.1038/s41598-022-07814-z

**Published:** 2022-03-08

**Authors:** Laura Llamosas-Falcón, Germán Sánchez-Díaz, Elisa Gallego, Ana Villaverde-Hueso, Greta Arias-Merino, Manuel Posada de la Paz, Verónica Alonso-Ferreira

**Affiliations:** 1 Centre for Addiction and Mental Health, Institute for Mental Health Policy Research, 33 Ursula Franklin Street, Toronto, ON M5S 2S1 Canada; 2 Department of Geology, Geography and Environmental Sciences, University of Alcala, 28801 Alcalá de Henares, Spain; 3 Centre for Biomedical Network Research On Rare Diseases (CIBERER), Instituto de Salud Carlos III, 28029 Madrid, Spain; 4 Department of Preventive Medicine, General University Hospital of Ciudad Real, 13005 Ciudad Real, Spain; 5 Institute of Rare Diseases Research (IIER), Instituto de Salud Carlos III, 28029 Madrid, Spain

**Keywords:** Neuromuscular disease, Epidemiology

## Abstract

Muscular dystrophies (MD) are a group of rare hereditary degenerative diseases. Our aim was to analyze the mortality pattern in Spain from 1981 to 2016 to assess the temporal trend and discern possible geographic differences using population-based data. Annual deaths related to MD were obtained from the National Statistics Institute with codes 359.1 of the ICD-9 (1981–1998) and G71.0 of the ICD-10 (1999–2016). Age-adjusted mortality rates were calculated and changes in mortality trends were identified. The standardized mortality ratios (SMR) and their respective 95% confidence intervals were calculated by district for 1999–2016. Smoothed SMRs and posterior probability were also assessed and then mapped to look for patterns or geographic distribution. All rates were expressed per 1,000,000 inhabitants. A total of 2,512 deaths (73.8% men) were identified. The age-adjusted mortality rates varied from 0.63 (95% CI 0.40–0.95) in 1981 to 1.51 (95% CI 1.17–1.93) in 2016. MD mortality showed a significant increase of 8.81% per year (95% CI 5.0–12.7) from 1981 to 1990, remaining stable afterwards. Areas with risk of death higher than expected for Spain as a whole were identified, not showing a specific regional pattern. In conclusion, the rising trend in MD mortality might be attributable to advanced improvements in diagnostic techniques leading to a rise in prevalence. Further research on the districts with the highest mortality would be necessary.

## Introduction

Muscular dystrophies (MD) are a group of rare hereditary degenerative diseases that share clinical characteristics highlighting the progressive muscle weakness in the affected patients^1,2^. It encompasses a series of congenital disorders such as Duchenne or Becker, pelvic girdle, distal, facioscapulohumeral, Emery-Dreifuss, ocular and oculopharyngeal, among others. These disorders are differentiated by the age at which the first symptoms appear, the severity of the symptoms, the inheritance pattern and the affected muscles pattern^1^. This determines the quality of life and mortality, being the cardiorespiratory failure one of the main causes of death^3^.

The worldwide prevalence of combined MD varies from 3.8 per 100,000 in Japan to 26.8 per 100,000 in Egypt, with a narrow range estimation of 19.8 to 25.1 per 100,000^4–6^, therefore it meets the criteria to be considered a rare disease. In addition, the prevalence of the most common disorder of the group, Duchenne muscular dystrophy (DMD), has been estimated in 3 cases per 100,000 persons (7 cases per 100,000 in men)^7^. In Europe, the prevalence is generally higher compared to the general population^8–10^. Regarding the situation in Spain, the prevalence of MD is still unknown.

There are some clinical and epidemiological surveillance networks such as MD STARnet (patient registry in the United States)^2^, the TREAT-NMD network (guaranteeing tools for the development of new therapies and linking up MD registries)^11,12^ and the registry of patients NMS-ES in Spain. Despite the existence of these registries, there is a lack of population-based information on the temporal trend and geographic variations they might have.

Currently, there is an important lack of epidemiological studies regarding rare diseases thus efforts should be put to investigate them^13,14^. Epidemiological studies of rare diseases are necessary to increase public awareness and to develop the bases of the knowledge for clinicians dealing with these particular patients. The scientific information given is mostly obtained from a clinical sight rather than a population view. So, using a population-based analysis would fill the gap that is currently prevailing in the epidemiology of rare diseases^13,15^. As studied before in other rare diseases (e.g.^16,17^), descriptive epidemiological studies provides the basis for future analysis to determine the causes of the variability in the trend. In the case of MD, with a cause of development mostly genetic, geographic studies could help to discover patterns of high-risk areas that were unknown. They could contribute to possibly identify the prevalence in the carrier and improve the genetic counseling of the families. In addition, mortality studies are useful to study the trend by reporting demographic changes and suggesting the progression of the disease and factors associated with the outcome. There are no nationwide studies on mortality attributed to MD in Spain, therefore, our objectives were analyzing the mortality patterns over several decades, assessing whether there is any temporal trend, and discerning possible geographical differences in the risk of death due to MD.

## Material and methods

The registered annual deaths due to MD in the period 1981–2016 were obtained from the National Statistics Institute (NSI) of Spain. Deaths were identified by reference to the International Classification of Diseases (ICD), with the codes 359.1 of the Ninth Revision (ICD-9, period 1981–1998) and G71.0 of the Tenth Revision (ICD-10, period 1999–2016).

Data on age, sex, year of birth and death, and place of residence were considered. Annual data of the Spanish population, broken down by sex, age and place of residence were also obtained from NSI, which we used to calculate the crude mortality rates and the age-adjusted mortality rates for men, women and both sexes using the European standard population as a reference. All rates were expressed per 1,000,000 inhabitants. The temporal trend was evaluated using the Joinpoint regression model, useful for identifying and describing temporal changes and offering a clear image of the trend over long periods^18^.

For each of the geographic units (districts), standardized mortality ratios (SMRs) were calculated in the period 1999–2016 using the Spanish population as a reference. We used an approximation of the exact test by Byar^19^ to calculate the SMRs and their confidence interval at 95%. The SMRs were subsequently smoothed taking into account the information related to the adjacent geographic units, according to the model proposed by Besag, York and Molliè^20^. Finally, the posterior probability (PP) of the smoothed-SMR, a Bayesian indicator, was calculated, where values show significantly higher (PP > 0.80) or lower (PP < 0.20) risk of death due to MD than expected for Spain as a whole.

We designed and conducted an observational, retrospective, descriptive study. All statistical analyses were performed using the SPSS v15 (IBM Corporation, Chicago, IL, USA), EPIDAT v4.2 (General Directorate of Public Health, Galicia, Spain), Joinpoint v4.5.0.1 (National Cancer Institute, Bethesda, MD, USA) and R-INLA (Norwegian University of Science and Technology, Trondheim, Norway) computer software programs, while ArcGIS 10.2 (ESRI, Redlands, CA, USA) was used for cartographic representation purposes.

### Ethics approval and consent to participate

The study was conducted in accordance with the Declaration of Helsinki, and the protocol was approved by the Ethics Committee of the Instituto de Salud Carlos III (CEI 50/2013).

## Results

A total of 2,512 MD-related deaths were identified in Spain during the period 1981–2016 (73.8% men). The mean age of death was 43 ± 24 years, being this value 37 ± 23 years in men and 59 ± 21 years in women (p < 0.001).

**Figure 1:**
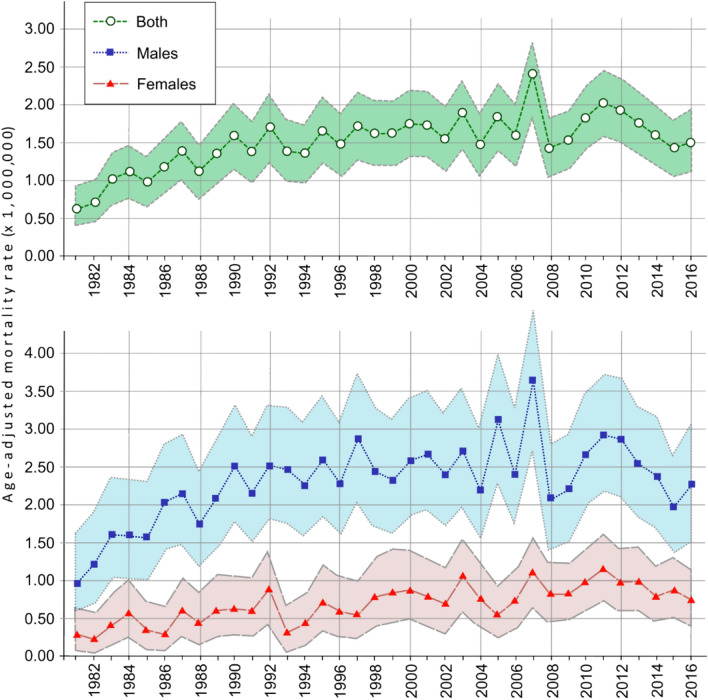
Age-adjusted mortality rates due to Muscular dystrophies from 1981 to 2016 in Spain. Shadows represent Confidence Intervals 95%.

Analyzing the time trend, the crude mortality rate in both sexes varied from a minimum of 0.63 per 1,000,000 inhabitants in 1981 to a maximum of 2.46 per 1,000,000 inhabitants in 2007. As shown in Fig. 1, the age-adjusted rates varied from 0.63 (95% CI 0.40–0.95) per 1,000,000 inhabitants in 1981 to 1.51 (95% CI 1.17–1.93) per 1,000,000 inhabitants in 2016. According to the Joinpoint analysis, an annual increase of 8.81% (95% CI 5.0–12.7; p < 0.05) is observed between 1981 and 1990, while from 1990 onwards the trend did not present a statistically significant fluctuation. By sex, this trend was similar in men showing an increase of 8.89% (95% CI 5.0–12.9; p < 0.05) between 1981 and 1990, while in women an increase of 2.95% (95% CI 2.1–3.8; p < 0.05) throughout the period 1981–2016 is observed in the age-adjusted mortality rates.

**Figure 2:**
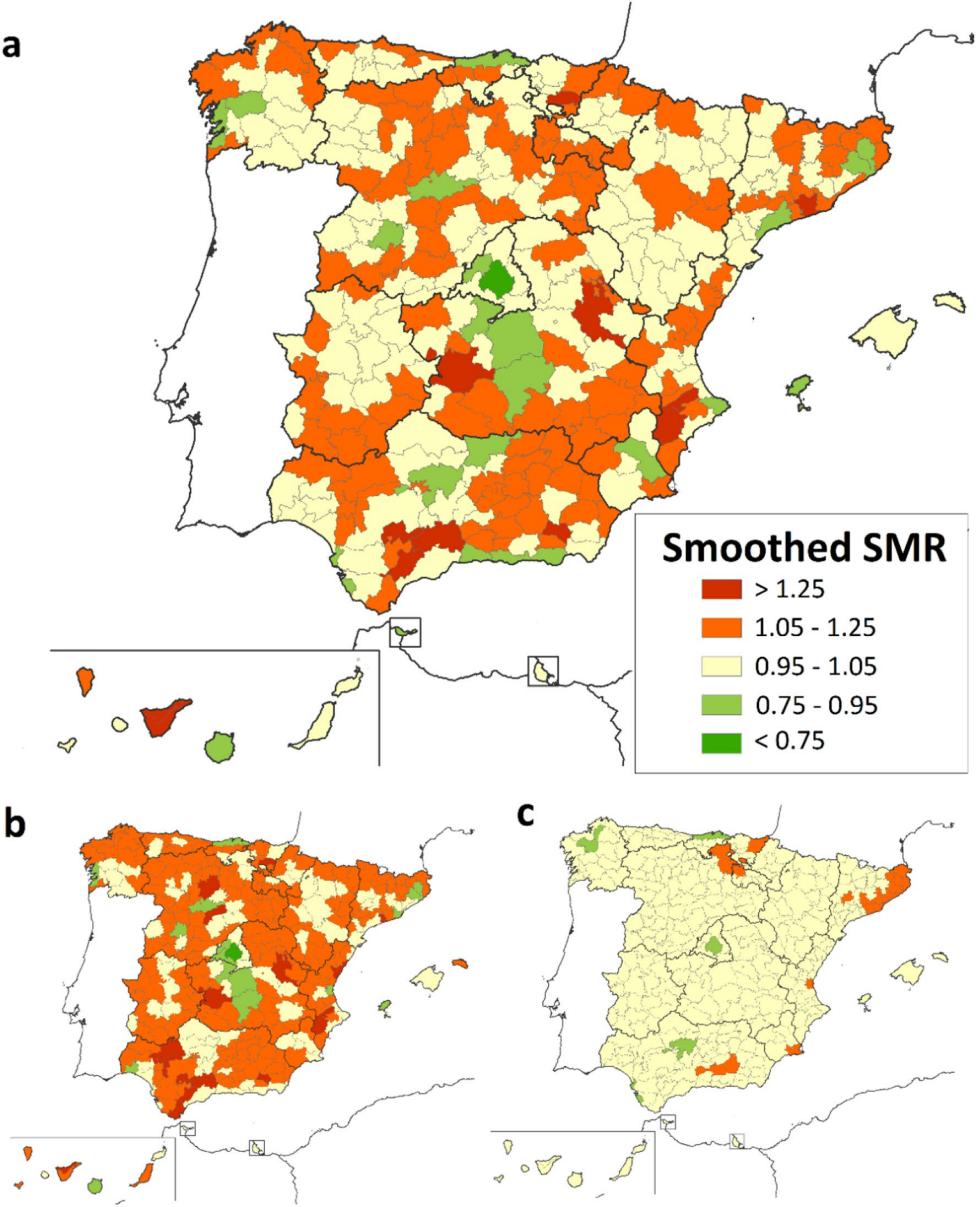
Smoothed-SMRs for Muscular dystrophies (1999–2016): (**a**) Both sexes (**b**) Males (**c**) Females.

**Figure 3:**
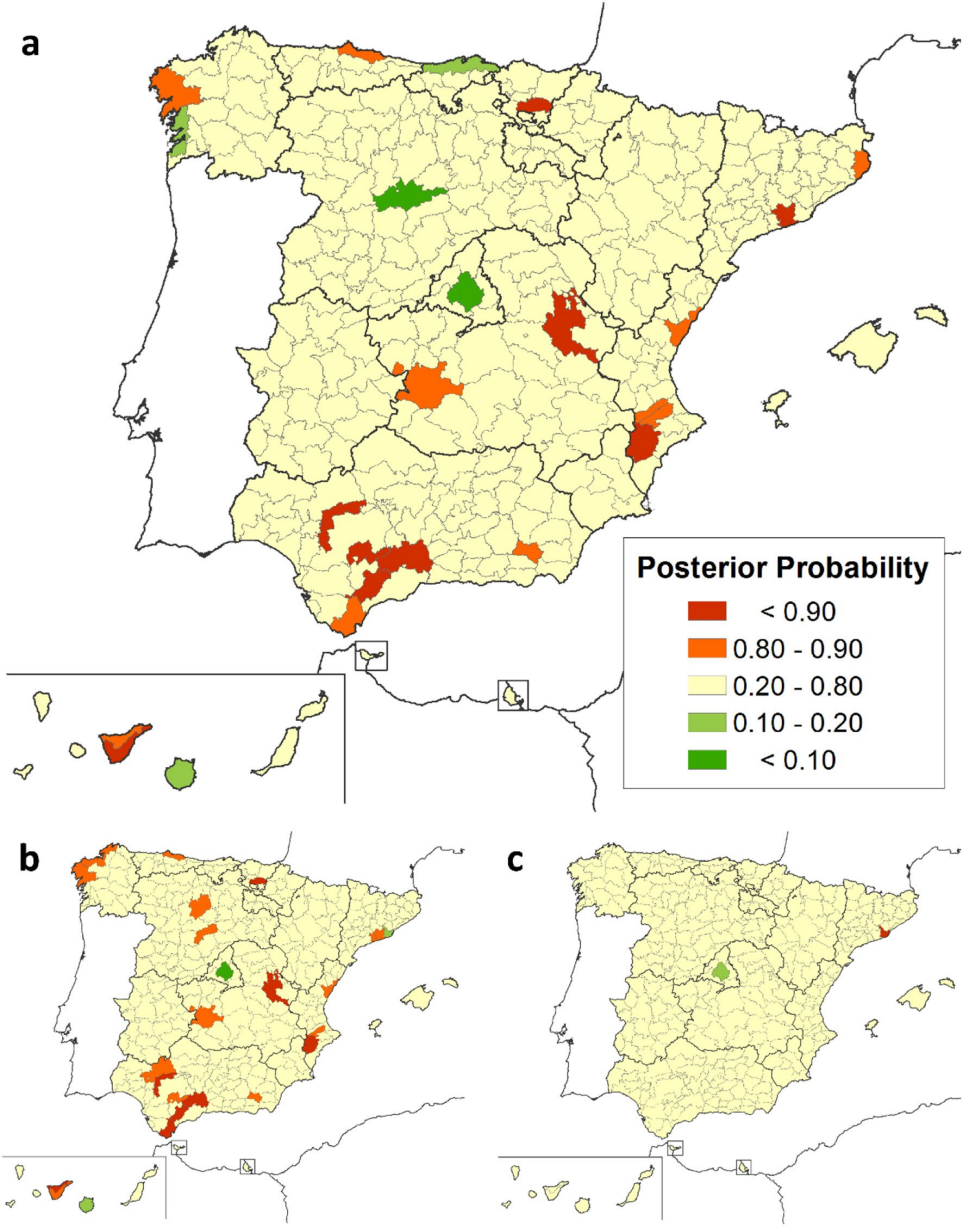
Posterior probability for Muscular dystrophies’ risk of death (1999–2016): (**a**) Both sexes (**b**) Males (**c**) Females.

Regarding the geographical analysis, the risk of death for both sexes combined was lower than expected in some districts of the provinces of Illes Balears, Valladolid, Cantabria and Madrid, and higher than expected in 12 different districts (Table 1). Among males, the SMR was lower than expected in one district of the province of Madrid and higher than expected in 13 different districts, while among females, only 4 districts in the provinces of Barcelona, Guipúzcoa, La Rioja and Badajoz were identified as having a higher-than-expected SMR. Taking into account the information related to the adjacent districts, smoothed-SMRs showed again more variability in the risk of death due to MD in men than in women (Fig. 2). When calculating the PP (Fig. 3), we identified for both sexes, 5 districts with a risk of death significantly lower than expected (PP < 0.20), while it was higher than expected in 19 districts (PP > 0.80). In the case of women, higher risk was identified in 1 district of Catalonia and lower risk in 1 district of Madrid. In men, 3 districts showed lower mortality, but 22 districts have been identified with higher than expected risk of death belonging to the Autonomous Communities of Andalusia, Castile La Mancha, Valencian Community, Catalonia, Castile and Leon, Basque Country, Asturias, Galicia and the Canary Islands.

**Table 1:** Statistically significant standardized mortality ratio (SMR) by districts in 1999–2016, 95% confidence intervals, global and by sex.

Risk	Location	Province	Districts	Both Gender SMR (95% IC)	Male SMR (95% IC)	Female SMR (95% IC)
Low Risk	N	Cantabria	Costera	0.52 (0.24—0.99)		
	C	Valladolid	Centro	0.36 (0.12—0.85)		
	C	Madrid	Área Metropolitana	0.67 (0.54—0.81)	0.64 (0.50—0.82)	
	E*	Illes Balears	Ibiza	0 (0—0.92)		
High Risk	N	Guipúzcoa	Guipuzcoa			1.92 (1.07—3.16)
	N	Álava	Llanada Alavesa	2.18 (1.29—3.44)	2.27 (1.21—3.88)	
	N	Cantabria	Tudanca—Cabuérniga	10.36 (1.16—37.42)		
	NE	Barcelona	Bajo Llobregat			1.58 (1.16—2.11)
	NE	Barcelona	Penedés	1.85 (1.01—3.10)		
	NE	La Rioja	Rioja Alta			7.33 (1.97—18.77)
	C	Cuenca	Serranía Media	4.52 (2.16—8.30)	5.24 (2.26—10.32)	
	C	Ciudad Real	Montes Norte	4.53 (1.46—10.58)	5.15 (1.38—13.18)	
	C	Soria	Campo de Gómara	5.90 (1.18—17.22)		
	C	Toledo	Montes de Navahermosa	6.83 (1.37—19.97)		
	W	Badajoz	Puebla de Alcocer			13.65 (2.74—39.90)
	E	Alicante	Vinalopó	1.87 (1.09—2.99)	2.15 (1.78—3.61)	
	E	Valencia	Valle de Albaida		2.79 (1.02—6.07)	
	S	Málaga	Norte o Antequera	3.05 (1.62—5.22)	3.29 (1.57—6.05)	
	S	Málaga	Serranía de Ronda	4.60 (2.10—8.72)	5.07 (2.03—10.44)	
	S	Almería	Río Nacimiento	11.23 (3.02—28.76)	16.33 (4.39—41.81)	
	SW	Cádiz	Campo de Gibraltar		2.16 (1.15—3.70)	
	SW	Sevilla	La Vega		1.49 (1.05—2.05)	
	SW	Sevilla	Sierra Sur	3.60 (1.55—7.10)	3.13 (1.01—7.31)	
	SW	Sevilla	Sierra Norte		3.44 (1.11—8.02)	
	SW*	Santa Cruz de Tenerife	Norte de Tenerife		1.90 (1.10—3.05)	

* Island territories (Canary and Balearic Islands).

C = Centre; E = east; N = north; NE = northeast; NW = northwest; S = south; SE = southeast; SW = southwest; W = west.

## Discussion

Our study is the first nationwide study that analyzes the temporal and geographic patterns of mortality due to MD across a 36-year period in Spain. A significant increase in mortality has been detected until 1990, as well as differences in the risk of death throughout the Spanish territory.

MD have different inheritance patterns. DMD is the most prevalent disorder and it is X-linked which implies that women are the carriers with little symptomatic expressiveness while men are the symptomatic patients with the most severe forms and early mortality^21^. This characteristic explains the differences by sex in number of registered deaths and mean ages of mortality. In some studies no evidence has been found that carriers of recessive genes have a reduced mortality compared to women in the general population, and it is important to maintain close monitoring for possible cardiac complications that could be develop^22,23^.

Great advances have been made in early diagnosis and treatment which improved the survival and quality of life of MD patients. In the 80 s and 90 s, there used to be a delay in diagnosis and consequently the treatment was initiated when the disease was advanced^24–26^. Historically, due to clinical suspicion, a protein analysis was performed by muscle biopsy, but currently, genetic tests are being used^27,28^. Garcia et al. conducted a study in Andalusia (Spain) using a recent diagnostic technique useful for the study of index cases and possible carriers^29^: MLPA (multiple ligand-dependent probe amplification), concluding that it is possible to offer adequate genetic counseling without the need for a muscle biopsy. Carrier confirmation affects family planning in addition to the possible early establishment of a close follow-up of such individuals since the mutation and affectation of the case can be identified early. On the other hand, there is also evidence that some affected patients did not seek medical assistance due to little hope of cure and perception that there was little benefit from health services^30^. Therefore, we can suspect that the observed increase in mortality may be due to an improvement in the diagnosis of this group of disorders, which correctly identifies MD patients including the ones with late diagnosis and a higher probability of death.

To date, there is no cure for MD and the treatment is multidisciplinary and symptomatic. In recent years, the different targets that improved quality of life and prolonged survival have been studied^31,32^. Since 1980, MD patients had been treated with corticosteroids as standard outpatient treatment. The mechanism of action is unknown, but it is related to the anti-inflammatory and immunosuppressive properties as well as by interaction with various genes. In addition, it increased muscle strength and delayed the progression of cardiac and respiratory dysfunction^31^. However, its prolonged use also involves side effects that can decrease survival and increase complications related to immunosuppression^33^.

One of the main causes of death reported in patients with MD is cardiorespiratory failure^34^. Until the 90 s decade, patients died from respiratory complications, but since ventilatory assistance was introduced as the basis of treatment, the trend changed and cardiac complications, especially cardiomyopathies developed as a result of muscle involvement, became the main cause of death^35,36^. According to Kieny et al., deaths from cardiac complications raised from 8 to 40% in the period 1980–2011^35^. The increase in the frequency of cardiomyopathies and the decrease in deaths from pulmonary complications are consistent with improvements in treatment which reduced respiratory failure and increased the age of death. The important advance in respiratory ventilation therapies has triggered a decrease in mortality^37^. A mean survival of around 20 years has been reported in patients who were not treated with respiratory ventilation, in contrast to the mean of between 25 and 30 years in patients with respiratory ventilation as a base treatment^35,38,39^. In recent decades, new treatment options have emerged including corrective surgery, treatment directed at specific mutations and experimental clinical trials. Physiotherapeutic rehabilitation has become important in both respiratory and muscular levels, as well as other targets of treatment such as bone, metabolism, gastrointestinal and nutritional^31^.

Regarding the geographic analysis, we have identified areas where the mortality due to MD is higher than expected for Spain as a whole. These districts are distributed and dispersed heterogeneously throughout the territory and they do not fit any pattern. Various international studies have not found a specific geographic pattern either; there is usually a higher prevalence of MD in Europe compared to the world, but no regional differences have been defined^8,36^. In Spain, a multicenter study was carried out in the period of 2007 and 2014 analyzing diagnosed cases of DMD in the main laboratories of the country, showing a heterogeneous geographical distribution. However, in this study they indicated that they collected less cases than expected, probably showing difficulties for these patients to access diagnostic reference centers^40^. On the other hand, in Navarra (Spain) the prevalence and genetic characterization of hereditary muscle diseases were studied, including dystrophic myopathy (not included in our analysis), identifying a dispersion of cases^41^.

Mortality studies are needed in the epidemiological field of rare diseases to fill the gaps in the scientific literature about how they behave overtime and geographically. Also, evaluating mortality in large registries (such as TREAT-NMD) provides access to valuable patient data which could improve the specific knowledge of MD and the association with their genetic etiology, as well as the management and treatment^42^. Overall, the results presented in our study could help the scientific community to understand how this group of rare diseases have evolved in three decades in Spain. This finding could be used to form the epidemiological bases of MD and complement the information provided by other registries of rare diseases, making our findings useful when comparing ourselves with other countries. In addition, our results are relevant to monitor MD mortality, support health planning in specific areas consider as on-risk and be the basis for subsequent studies which identify the determinants of the geographic pattern detected.

The present study has several limitations. We selected annual deaths based on the registry of the respective ICD-9 and ICD-10 code on their death certificate. The death registry of MD should be based on the underlying cause of death, because of its chronicity, and not necessarily on the immediate cause of death. In consequence, we could be facing a possible underestimation of the real results when the cases are not register correctly. In addition, the identification of MD as cause of death by ICD-9 and ICD-10 codes does not allow the study to be more specific in analyzing each of the different disorders belonging to this group of diseases. Despite these limitations, the strength of our study is that following a homogeneous, standardized and continuous methodology, we have analyzed the mortality officially due to MD in Spain over a long period, also serving as the basis for next studies.

In conclusion, in recent decades, efforts have been focused on studying in depth the set of MD, improving early diagnosis techniques as well as therapeutic targets aimed at prolonging the survival of these patients. This is the first nationwide study that analyzed the temporal-spatial variability of mortality due to MD across decades in Spain. The identification of areas with higher mortality than expected has an important implication for medical care planning and highlights the need to create a national surveillance network due to the wide regional variation that we have observed. Future studies should focus on analyzing the risk in the districts with the highest mortality rates and their determining factors in order to appropriately allocate resources to improve health care for this population.

## Data Availability

The datasets generated and/or analysed during the current study are available in the National Statistics Institute (NSI) repository, at https://www.ine.es/.

## Abbreviations

MD: Muscular dystrophy
DMD: Duchenne muscular dystrophy
NSI: National Statistics Institute
ICD: International Classification of Diseases
SMRs: Standardized mortality ratios
PP: Posterior probability
95% CI: 95% Confidence interval
